# Multistrain Probiotic Supplementation Combined with a Standardized Diet Did Not Significantly Affect Exercise Performance or Inflammatory Responses in Male Endurance Runners: A Randomized Controlled Trial

**DOI:** 10.3390/nu18152484

**Published:** 2026-08-01

**Authors:** Katarzyna Jagłowska, Marcin Folwarski, Maciej Chroboczek, Marta Potrykus, Mariusz Kaczmarczyk, Karolina Skonieczna-Żydecka, Jan Jacek Kaczor

**Affiliations:** 1 Division of Physiology, Gdansk University of Physical Education and Sport, 80-336 Gdansk, Poland; katarzyna.jaglowska@awf.gda.pl (K.J.); maciej.chroboczek@awf.gda.pl (M.C.); 2 Division of Bioenergetics and Exercise Physiology, Medical University of Gdansk, 80-211 Gdansk, Poland; 3 Department of Clinical Nutrition and Dietetics, Medical University of Gdansk, 80-211 Gdansk, Poland; marcinfol@gumed.edu.pl (M.F.); martapotrykus@gumed.edu.pl (M.P.); 4 Department of Biochemical Science, Pomeranian Medical University in Szczecin, 71-460 Szczecin, Poland; mkariush@gmail.com (M.K.); karolina.skonieczna.zydecka@pum.edu.pl (K.S.-Ż.); 5 Department of Animal and Human Physiology, Division of Bioenergetics, Faculty of Biology, University of Gdansk, 80-309 Gdansk, Poland

**Keywords:** exercise metabolism, cytokines, creatine kinase, aerobic capacity, inflammation, gut microbiota

## Abstract

**Background:** Gut microbiota may influence metabolic and inflammatory responses to exercise through the gut–muscle axis, and probiotic supplementation has been proposed to support adaptation and recovery in endurance athletes. However, evidence in trained populations is inconsistent and is confounded by variability in diet, probiotic strains, dose, and intervention duration. We tested whether a multistrain probiotic, administered against a fully standardized diet, would affect exercise performance and inflammatory, metabolic, and muscle-damage responses. **Methods:** In this randomized, double-blind, placebo-controlled trial, 30 trained male long-distance runners were randomized and 27 completed the study (probiotic [PRO], *n* = 13; placebo [PLA], *n* = 14). All participants followed a standardized, normocaloric meal-box diet for four weeks. Aerobic capacity (peak oxygen uptake, VO_2_peak; primary outcome) was assessed by an incremental treadmill test and anaerobic performance by a 30 s Wingate test, before (PRE) and after (POST) supplementation. Interleukin-6 (IL-6), tumor necrosis factor-α (TNF-α), creatine kinase (CK), and lactate (LA) were measured around exercise. Outcomes were analyzed with linear mixed-effects models; the primary estimand was the group × phase interaction. **Results:** VO_2_peak did not differ between groups over time (PLA 55.00 ± 7.60 → 57.33 ± 8.41; PRO 55.40 ± 7.85 → 53.14 ± 5.95 mL·kg^−1^·min^−1^; group × phase −3.5 mL·kg^−1^·min^−1^, 95% CI −7.0 to −0.1; nominal *p* = 0.044, not significant after FDR correction, adjusted *p* = 0.707). No group × phase or group × phase × sampling-time interaction was significant for IL-6, TNF-α, LA, or CK (all interaction *p* > 0.15), indicating that supplementation did not modify the exercise-induced response of any biomarker; exercise itself robustly increased IL-6, TNF-α, and LA in both groups (*p* < 0.001). **Conclusions:** In trained male endurance runners consuming a standardized diet, four weeks of multistrain probiotic supplementation did not significantly affect aerobic or anaerobic performance, LA response, muscle-damage markers, or circulating inflammatory cytokines compared with placebo. Adequately powered trials with prespecified primary endpoints and direct microbiome assessment are needed.

## 1. Introduction

Probiotic supplementation has attracted growing interest in sports nutrition as a means of supporting intestinal function, modulating exercise-induced inflammation, and potentially enhancing performance and recovery in athletes [1]. Several trials in endurance and mixed-sport populations have reported favorable effects, including reduced circulating cytokines and improved intestinal barrier markers [2] and in some cases improvements in aerobic capacity or time to exhaustion [3,4]. However, the evidence is inconsistent: comparably designed studies have found no effect on inflammatory markers, lactate (LA) metabolism, or classical performance indices [5,6,7,8]. Systematic reviews conclude that any ergogenic or anti-inflammatory effect is small and strongly dependent on the specific strains, dose, intervention duration, athletic population, and exercise protocol [9]. A recurring limitation is that diet—the single most powerful determinant of gut microbial composition and activity [10,11]—is rarely controlled, leaving it unclear whether reported effects reflect the probiotic itself or concurrent nutritional variation.

The biological rationale for these interventions rests on the gut–muscle axis, a bidirectional signalling network through which microbial metabolites such as short-chain fatty acids (SCFAs) interact with host metabolism, inflammatory pathways, and skeletal muscle function [12,13,14]. This rationale is particularly relevant to endurance runners, in whom high-volume training and prolonged reductions in splanchnic perfusion can compromise intestinal barrier integrity, increase permeability, and promote translocation of bacterial components and low-grade systemic inflammation [15,16]. Such stress may shift microbial composition toward a less favourable, more pro-inflammatory profile [17]. Certain probiotic strains have been proposed to counteract these processes by strengthening the epithelial barrier and attenuating cytokine responses [2], and LA-utilizing taxa may enhance LA clearance and substrate use during exercise [1].

Despite this rationale, it remains unknown whether probiotic supplementation exerts measurable physiological effects in well-trained endurance athletes when dietary intake is fully standardized, thereby isolating the intervention from its main confounder. To our knowledge, no trial has evaluated a multistrain probiotic against a fully controlled, individually prescribed meal-box diet in this population. The specific multistrain formulation (Sanprobi Active & Sport), daily dose, and four-week period used here were selected to match our previous randomized trial in male mixed martial arts athletes, in which the same strains, dose, and four-week period—there co-administered with vitamin D_3_—were associated with improvements in selected performance markers [18]. Because that regimen included vitamin D_3_, the earlier findings serve as a design analogue rather than evidence of an independent effect of probiotic suplementation. Applying the matched probiotic regimen (without vitamin D_3_) in an endurance population, while adding strict dietary standardization, allows a more isolated evaluation across contrasting exercise phenotypes.

We therefore conducted an exploratory (preliminary), randomized, double-blind, placebo-controlled trial—not powered as a confirmatory efficacy trial—to examine whether four weeks of multistrain probiotic supplementation, combined with a standardized diet, affects performance, inflammatory, metabolic, and muscle-damage responses in trained male long-distance runners. We hypothesized that, relative to placebo, probiotic supplementation would improve aerobic and anaerobic performance (higher VO_2_peak, time to exhaustion, and power output), attenuate the post-exercise inflammatory response (lower IL-6 and TNF-α), reduce the post-exercise blood lactate response, and lower exercise-induced creatine kinase activity.

## 2. Materials and Methods

### 2.1. Study Design

This study was designed as a preliminary, randomized, double-blind, parallel, placebo-controlled clinical trial including competitive long-distance runners. Because of the limited sample size and the exploratory character of the intervention, the study was intended to generate preliminary evidence rather than to provide confirmatory conclusions. For a period of four weeks, all participants received a standardized box-diet and either a probiotic supplement or a placebo, depending on group assignment.

Participants were randomized in a 1:1 ratio using a simple randomization procedure generated in Microsoft Excel and prepared by an independent researcher who was not involved in participant recruitment, eligibility assessment, outcome measurements, or statistical analysis. Allocation concealment was ensured by using identical, coded containers prepared according to the randomization list. Participants were enrolled and assessed by the study investigators, who assigned the next available coded container in sequential order without knowing the treatment allocation. Blinding was maintained throughout the study and was only lifted after completion of all statistical analyses. Measurements were taken at two time points: before the intervention (PRE) and immediately after the four-week supplementation period (POST). The same experimental sequence was applied at both time points.

The study was conducted in accordance with the Declaration of Helsinki [19] and approved by the Independent Bioethics Committee for Scientific Research at the Medical University of Gdańsk (No. NKBBN/643/2019-2020), approved on 14 January 2020. All participants enrolled in the study were fully informed about the study’s product and protocol and provided a signed informed consent form before any assessment or intervention. The trial was registered retrospectively at ClinicalTrials.gov (NCT07411482; first submitted 31 December 2025, first posted 13 February 2026), after completion of participant enrolment.

**Figure 1:**
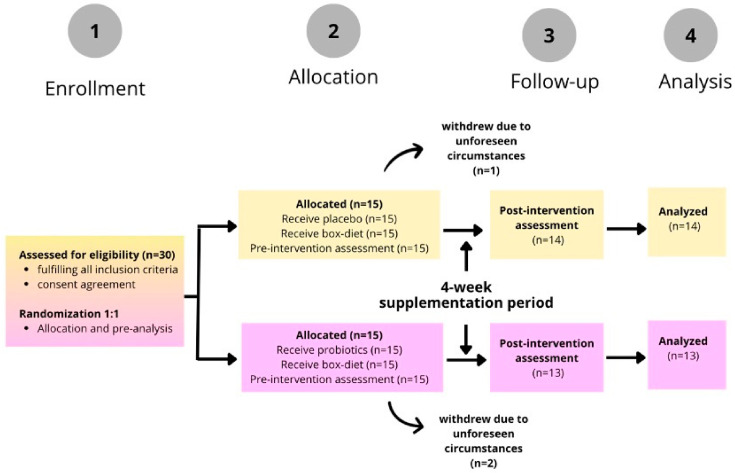
CONSORT participant flow diagram: assessed for eligibility, randomized (*n* = 30), allocated to probiotic and placebo, followed up (3 withdrawals for personal reasons), and analyzed (probiotic *n* = 13; placebo *n* = 14).

However, the primary outcome (VO_2_peak), secondary outcomes, and the a priori sample-size calculation had been specified before enrolment in both the bioethics-approved study protocol and the funded National Science Centre grant application (2021/41/N/NZ4/02364). The gut microbiota outcome, originally registered as a co-primary endpoint, is reported separately in a dedicated manuscript. This manuscript was prepared in accordance with the CONSORT 2010 statement for randomized parallel-group trials [20]. The study took place in Gdańsk, Poland, from September to December 2023. The study design is illustrated in Figure 1.

### 2.2. Participants

The study enrolled adult male long-distance runners. Recruitment and all assessments were conducted at a single site (Gdansk University of Physical Education and Sport, Gdańsk, Poland); participants were recruited from local running clubs and by public advertisement. Inclusion criteria required participants to be 18 years of age or older, to engage in a minimum of five structured running sessions per week, to have at least three years of regular endurance training experience, and to maintain an average marathon pace of 10.5–14 km/h. Eligible participants also had to possess prior competitive experience, defined as the completion of at least three half-marathons or full marathons.

Exclusion criteria included age below 18 years, antibiotic therapy during the past three months, diagnosed inflammatory bowel disease, chronic musculoskeletal injuries within the preceding six months, and a history of heart failure or structural cardiac defects. Enrolment was restricted to male runners a priori to reduce sex- and menstrual-cycle-related variability in inflammatory, metabolic, and performance responses, which could obscure a modest intervention effect in a small sample; this restriction limits generalizability to female athletes and is addressed in the Limitations. All participants provided written informed consent after receiving comprehensive information regarding the study protocol and procedures. A total of 30 participants were randomized. Three participants did not complete the full protocol because of personal reasons. Therefore, 27 participants were included in the final per-protocol analysis: 13 in the probiotic group and 14 in the placebo group.

The a priori target sample size was determined using G*Power 3.1.9.6 for Mac OS. The calculation was anchored on the post-intervention reduction in TNF-α reported by Townsend et al.; assuming a one-sided test at α = 0.05 and a large effect size (Cohen’s d = 0.82), a total of 40 participants (1:1 allocation) was required to provide 80.4% power. Recruitment yielded 30 randomized participants, of whom 27 completed the protocol (PRO *n* = 13; PLA *n* = 14); the planned target of 40 was not reached owing to recruitment feasibility and participant withdrawal, which we address as a limitation. Analyses followed a per-protocol (complete-case) approach: the three participants who withdrew before the POST assessment were not included, and no imputation was performed. The mixed models retained all available observations under a missing-at-random assumption, so a participant with an isolated missing measurement still contributed available data to the relevant model. Given the achieved sample-and because the original calculation assumed a large effect under a one-sided test—the study was adequately powered only for large effects, and non-significant results are interpreted as an absence of evidence rather than evidence of absence. As the a priori calculation was specified one-sided in the direction of a probiotic benefit, the corresponding two-sided power at the same effect size and sample size is lower; we also note that the only nominally significant signal (the VO_2_peak group × phase interaction) was in the direction opposite to the hypothesized benefit. At the design stage, the calculation was anchored on TNF-α because, at the design stage, it offered the most reliable available prior effect estimate for probiotic supplementation in athletes [7]; we note that this estimate derived from a different athletic population and probiotic formulation, which is a further reason to regard the present trial as exploratory; VO_2_peak was designated the primary performance endpoint, so under the same sample size and α only a large VO_2_peak effect would be detectable—consistent with the study’s exploratory framing.

### 2.3. Intervention

The probiotic supplement contained lyophilized bacterial strains with documented beneficial effects on host health: Bifidobacterium lactis W51, Levilactobacillus brevis W63, Lactobacillus acidophilus W22, Bifidobacterium bifidum W23, and Lactococcus lactis W58. These were combined with maize starch, maltodextrin, plant proteins, and a hydroxypropyl ethylcellulose coating. The formulation is commercially available as Sanprobi^®^ Active & Sport (Szczecin, Poland) and was standardized to 2.5 × 10^9^ CFUs per gram, ensuring at least 500 million CFUs per capsule. The product underwent verification using pharmacopeial culture techniques, and CFU counts were guaranteed until the end of shelf life, confirmed under controlled climatic conditions. Capsules were stored at room temperature in a dry place before distribution. Participants were instructed to take four capsules daily with meals, two in the morning and two in the evening providing a total daily dose of 2 × 10^9^ CFU in the probiotic group. Placebo capsules were identical in appearance and excipients (maltodextrin and plant proteins) but contained no live bacteria. Mean capsule adherence was 92% in the probiotic group and 88% in the placebo group, assessed by capsule count. No serious adverse events were reported. The manufacturer of the probiotic product had no role in the study design, participant recruitment, randomization, data collection, laboratory analyses, statistical analysis, interpretation of the results, manuscript preparation, or the decision to submit the manuscript for publication.

To minimize variability in dietary influence on gut microbiota, all participants received a standardized meal-box diet (Body Chief, Poznań, Poland) for the entire intervention period. Individual energy requirements were calculated by a professional dietitian based on body mass and habitual training load, and the mean prescribed energy intake was approximately 3300 kcal/day. The diet was designed to provide comparable nutritional conditions across participants, with a relatively consistent macronutrient distribution, including approximately 1.6 g of protein/kg body mass and 5 g of carbohydrates/kg body mass per day. The diet was not specifically designed to increase fiber intake or fermented food consumption; instead, it was standardized to provide a comparable dietary pattern, as well as similar energy and macronutrient intake, across participants during the intervention. Adherence to the dietary protocol was verified during follow-up interviews conducted after the intervention.

### 2.4. Study Protocol

Participants attended two assessment sessions: the first at baseline, before the start of the intervention (PRE), and the second immediately after completing the four-week supplementation period (POST). Baseline measurements were carried out during the initial session, whereas follow-up assessments were performed the day after the intervention ended. At both time points, runners underwent body composition analysis, were interviewed regarding their dietary habits and completed training units as well as performed a standardized physical performance test. In addition, venous blood samples were collected before and after the performance test to determine the concentration of inflammatory markers: interleukin-6 (IL-6) and tumor necrosis factor-alpha (TNF-α), creatine kinase (CK) activity, and LA concentration. The primary outcome was peak oxygen uptake (VO_2_peak) measured during the incremental treadmill test. Secondary outcomes included time to exhaustion (TTE), respiratory exchange ratio (RER), minute ventilation (VE), Wingate-derived performance variables, and blood LA, CK, IL-6, and TNF-α concentration.

Participants were instructed to maintain their regular training routines while adhering to a standardized meal-box diet provided for the entire study period. To ensure consistency of training load, they were required to complete a minimum of five structured running sessions per week. Compliance was monitored through training diaries, in which participants recorded the duration of each session, heart rate (HR), and subjective ratings of perceived exertion (RPE). During the four-week intervention, training load did not differ significantly between the probiotic and placebo groups: training frequency 5.07 ± 0.62 vs. 5.31 ± 0.63 sessions/week (*p* = 0.335), mean session duration 82.71 ± 3.79 vs. 84.54 ± 3.45 min (*p* = 0.203), mean session heart rate 151.57 ± 1.91 vs. 152.62 ± 1.89 bpm (*p* = 0.167), and mean RPE 5.90 ± 0.24 vs. 5.96 ± 0.19 (*p* = 0.460).

### 2.5. Background Information

At the baseline assessment, participants provided detailed information on their health status, including previous or current injuries, medical conditions, and medication use, as well as dietary practices and training routines. During the follow-up visit, additional interviews were conducted to verify adherence to the standardized diet and training program, to record supplement intake, and to identify any intercurrent events such as infections, to ensure consistency, and to limit the influence of external factors.

### 2.6. Body Composition Analysis

Body composition was measured PRE- and POST-intervention using the InBody 720 analyzer (InBody Co., Ltd., Seoul, Republic of Korea), which applies multifrequency bioelectrical impedance [21]. Assessments were performed in the morning, in a fasted state, under standardized time-of-day and nutritional conditions. Participants stood upright, holding the hand electrodes and placing their feet on the designated sensors without body contact between limbs and torso. Before each measurement, demographic data (age, height, sex) were entered manually, and the electrodes were disinfected according to manufacturer instructions. Recorded outcomes included body weight, fat mass, and lean mass.

### 2.7. Blood Sampling and Biochemical Analyses

The timing of all blood and capillary samples relative to the exercise tests is summarized in Table 1; the identical schedule was used at the PRE and POST sessions. Venous blood was collected from the antecubital vein to assess plasma CK activity. Samples were taken at two standardized time points: before exercise (baseline) and 24 h following the sprint tests. After centrifugation at 3000× *g*, plasma was separated and stored at −80 °C until analysis. CK activity was determined using a kinetic assay at 37 °C with the RANDOX CK-NAC kit (CK522, Randox Laboratories Ltd., Crumlin, UK) according to the manufacturer’s protocol. Absorbance readings were performed on a Cecil CE9200 spectrophotometer (Cecil Instruments Ltd., Cambridge, UK), and CK activity was expressed in units per liter (U/L). Venous blood for IL-6 and TNF-α was collected at rest, 15 min after the aerobic test and 30 min after completion of the exercise protocol. The pro-inflammatory cytokines IL-6 and TNF-α were measured using commercially available ELISA kits (Diaclone SAS, Medix Biochemica Group, Besancon, France; catalog numbers 950.035.192 and 950.090.192, respectively) following the manufacturer’s protocols. The analytical sensitivity (lower limit of detection) was 0.81 pg/mL for IL-6 and 8 pg/mL for TNF-α. According to the manufacturer, the intra-assay and inter-assay coefficients of variation were <10% for both cytokines. Owing to the total number of samples, cytokine measurements were distributed across three microplates; however, all samples were analyzed within the same analytical series under identical laboratory conditions. All measurements were performed in duplicate.

Capillary blood samples were obtained from the fingertip by a trained researcher to evaluate lactate concentration. Sampling occurred prior to the tests, immediately after the aerobic test, 15 min post-aerobic test, and following the sprint test at 3, 15, 30, and 60 min. LA concentration was measured using the Lactate Scout 4 analyzer (EKF Diagnostics GmbH, Barleben, Germany) with dedicated test strips, following the manufacturer’s instructions; this device has demonstrated acceptable agreement with reference laboratory methods [22]. All analyses were performed in duplicate to ensure reliability.

### 2.8. Assessment of Physical Performance

All exercise tests were performed in the morning under controlled environmental conditions. Before testing, participants consumed a standardized breakfast provided by the investigators, consisting of white bread with jam and a banana. The meal was individually calculated to provide 0.9 g of carbohydrates per kilogram of body mass. Participants were instructed to avoid strenuous exercise for 24 h and caffeine or alcohol consumption for 12 h before testing. Aerobic capacity was assessed first because peak oxygen uptake, the primary outcome, requires a non-fatigued incremental test; anaerobic performance was then assessed after a standardized 15 min passive recovery so that residual fatigue was uniform across participants. This fixed order and recovery interval were applied identically at both the PRE and POST sessions and in both groups, ensuring that any potential carry-over effect was held constant and did not bias the within-participant, between-group comparison. Following the standardized 15 min passive recovery period, anaerobic performance was assessed on a cycle ergometer (Monark 884E Sprint Bike; Monark Exercise AB, Vansbro, Sweden).

Aerobic capacity was assessed using an incremental treadmill test performed to volitional exhaustion, adapted from a protocol previously used in endurance runners [23]. After a brief standing period on the treadmill to ensure stabilization of the measurement equipment, participants completed a standardized low-intensity warm-up, followed by a stepwise increase in exercise intensity until exhaustion. The incremental treadmill test started at 8 km/h and 0% incline, with running speed increased by 1 km/h every 1 min until volitional exhaustion. Test termination criteria included volitional exhaustion and the inability to maintain the required running speed despite verbal encouragement. Heart rate was continuously monitored (Polar Electro Oy, Kempele, Finland), and respiratory variables (VO_2_, VCO_2_, VE, and RER) were measured breath-by-breath using a calibrated metabolic cart (Oxycon Pro, Jaeger, Hoechberg, Germany). Peak oxygen uptake (VO_2_peak) was defined as the highest value recorded during the test.

Following a standardized 15 min passive recovery period, anaerobic performance was assessed on a cycle ergometer (Monark 884E Sprint Bike). The protocol included a 5 min warm-up at 100 W with 3–5 s maximal accelerations. After a 2 min rest, participants completed 30 s all-out Wingate anaerobic test, with resistance set to 7.5% of body weight [24]. Throughout the sprints, athletes were encouraged verbally to sustain maximal pedaling cadence.

### 2.9. Statistical Analysis

All analyses were performed in R version 4.4.0 using the lme4 package. Baseline participant characteristics were summarized descriptively as mean ± standard deviation or *n* (%) and compared between arms with independent-samples *t*-tests (Table 2). Distributional assumptions were assessed before modelling: normality of model residuals was examined using the Shapiro–Wilk test and quantile–quantile plots, and the appropriateness of the random-effects structure was checked by inspection of the estimated variance components and residual-versus-fitted plots; where residuals departed from normality, results were confirmed against a log-transformed analysis.

Longitudinal outcomes were analyzed with linear mixed-effects models. For single-measure outcomes assessed once per phase (VO_2_peak, time to exhaustion, respiratory exchange ratio, minute ventilation, and the Wingate-derived variables), the model included fixed effects for group (probiotic vs. placebo), phase (PRE vs. POST), and their group × phase interaction, with a participant-level random intercept (outcome~group × phase + (1|participant)). For outcomes sampled repeatedly within each phase (LA, IL-6, TNF-α, and CK), the model additionally included a fixed effect for sampling time and all two- and three-way interactions among group, phase, and sampling time, with nested random intercepts for participant and for participant-within-phase (outcome~group × phase × time + (1|participant) + (1|participant:phase)). Models were estimated by restricted maximum likelihood for the reporting of coefficients and confidence intervals; the statistical significance of the interaction terms was assessed by likelihood-ratio tests comparing nested models fitted by maximum likelihood. For the repeated-within-phase outcomes, the nested random-intercepts specification implies a compound-symmetric residual covariance across sampling times within a phase; alternative residual covariance structures (e.g., first-order autoregressive or unstructured) were not fitted.

The primary estimand was the group × phase interaction for single-measure outcomes and the group × phase × sampling-time interaction for repeated-within-phase outcomes; these quantify whether the change from PRE to POST differed between the probiotic and placebo arms. Results are reported as estimated between-group differences in change (regression coefficients) with 95% confidence intervals, exact *p*-values, and standardized effect sizes (Cohen’s d). Estimated marginal means were used for descriptive PRE/POST summaries; formal inference rested on the model interaction terms rather than on separate post hoc pairwise tests. To control the false-discovery rate across the family of outcomes, *p*-values were adjusted using the Benjamini–Hochberg procedure, and both nominal and FDR-adjusted values are reported for the primary outcome. Statistical significance was set at a two-sided α of 0.05.

## 3. Results

A total of 30 endurance runners were randomized, and 27 completed the full protocol (probiotic *n* = 13, placebo *n* = 14); three participants withdrew before the POST assessment for personal reasons unrelated to the intervention. Supplement adherence, assessed by capsule count, averaged 92% in the probiotic group and 88% in the placebo group. Adherence to the standardized meal-box diet was verified during follow-up interviews, and no major deviations from the prescribed dietary plan were reported. No serious adverse events related to the intervention were reported in either group. Actual training load during the intervention was comparable between groups (Table 2).

At baseline, there were no significant differences in anthropometric characteristics between the two groups. The average age was 42.08 ± 6.82 years in the probiotic (PRO) group and 39.43 ± 8.97 years in the placebo (PLA) group. Mean body weight was 76.76 ± 12.11 kg in PRO and 74.32 ± 8. kg in PLA, fat-free mass was 63.74 ± 7.71 kg and 62.34 ± 7.04 kg, and fat mass was 13.02 ± 5.57 kg and 11.99 ± 4.71 kg, respectively. Baseline cardiorespiratory fitness was comparable between the groups. VO_2_peak averaged 55.00 ± 7.60 mL·kg^−1^·min^−1^ in the PLA group and 55.40 ± 7.85 mL·kg^−1^·min^−1^ in the PRO group, while HRmax was 178.62 ± 8.54 bpm and 175.46 ± 9.57 bpm, respectively. Participant characteristics are summarized in Table 2.

### 3.1. Effects of Supplementation on Aerobic and Anaerobic Performance

The primary estimand—the group × phase interaction for VO_2_peak—was −3.5 mL·kg^−1^·min^−1^ (95% CI −7.0 to −0.1; nominal *p* = 0.044; Cohen’s d = −0.75), which did not remain significant after Benjamini–Hochberg correction (adjusted *p* = 0.707). No group × phase interaction was significant for RER (−0.015, 95% CI −0.081 to 0.051; *p* = 0.658), VE (0.41 L·min^−1^, 95% CI −10.8 to 11.6; *p* = 0.943), or TTE (−6.5 s, 95% CI −71.9 to 59.0; *p* = 0.847) (Table 3).

Similarly, no group × phase interaction was significant for total work (1.35 J·kg^−1^, 95% CI −3.07 to 5.77; *p* = 0.549; Cohen’s d = 0.23), mean power (0.064 W·kg^−1^, 95% CI −0.156 to 0.285; *p* = 0.568; Cohen’s d = 0.22), peak power (0.005 W·kg^−1^, 95% CI −0.331 to 0.342; *p* = 0.976; Cohen’s d = 0.01), or time to peak power (−0.63 s, 95% CI −1.84 to 0.58; *p* = 0.305; Cohen’s d = −0.39) (Table 3).

### 3.2. Effects of Supplementation on the Inflammatory State

**Figure 2:**
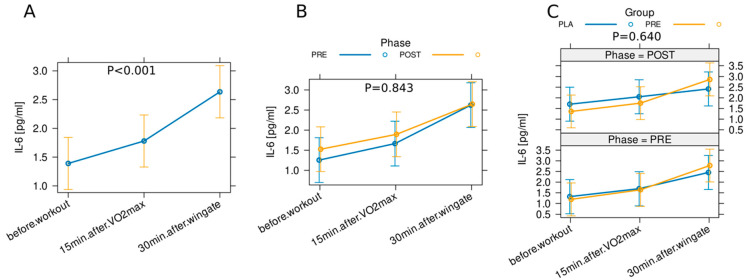
Interleukin-6 (IL-6) response to exercise before and after supplementation. Panels show the placebo (PLA) and probiotic (PRO) groups, including the overall response across sampling times (**A**), responses according to study phase (PRE vs. POST) (**B**), and the PLA and PRO groups within each study phase (**C**). Within each panel, IL-6 (pg/mL) is plotted at rest and at the post-exercise sampling times before (PRE) and after (POST) the four-week intervention. Points are estimated marginal means from the linear mixed-effects model (group × phase × sampling-time, with nested random intercepts for participant and participant-within-phase); error bars are 95% confidence intervals. The group × phase × sampling-time interaction was non-significant (χ^2^(2) = 0.19, *p* = 0.910), indicating that supplementation did not alter the exercise-induced IL-6 response.

**Figure 3:**
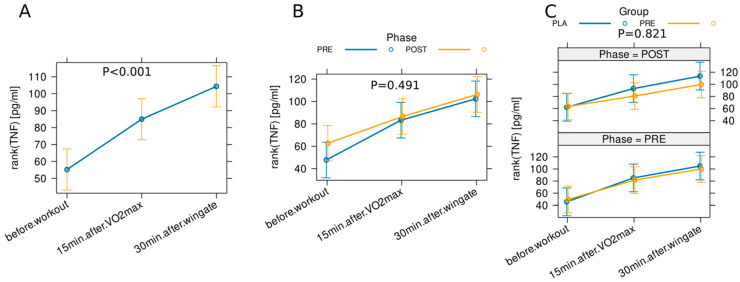
Tumor necrosis factor-α (TNF-α) response to exercise before and after supplementation. Panels show (**A**) the overall response across sampling times, (**B**) responses according to study phase (PRE vs. POST), and (**C**) the placebo (PLA) and probiotic (PRO) groups within each study phase. Within each panel, TNF-α (pg/mL) is plotted at rest and at the post-exercise sampling times before (PRE) and after (POST) the four-week intervention. Points are estimated marginal means from the linear mixed-effects model (group × phase × sampling-time, with nested random intercepts for participant and participant-within-phase); error bars are 95% confidence intervals. The group × phase × sampling-time interaction was non-significant (χ^2^(2) = 2.10, *p* = 0.350), indicating that supplementation did not alter the exercise-induced TNF-α response.

Neither the group × phase interaction (χ^2^(1) = 0.10, *p* = 0.754) nor the group × phase × sampling-time interaction (χ^2^(2) = 0.19, *p* = 0.910) was significant. The IL-6 responses are presented in Figure 2. IL-6 increased markedly after exercise in both groups at both study phases (*p* < 0.001), indicating that probiotic supplementation did not modify the exercise-induced IL-6 response. The TNF-α responses are presented in Figure 3. There was no group × phase (χ^2^(1) = 0.02, *p* = 0.902) or group × phase × sampling-time (χ^2^(2) = 2.10, *p* = 0.350) interaction, so the exercise-induced TNF-α response was unaffected by supplementation.

### 3.3. Effects of Supplementation on LA Blood Concentration

**Figure 4:**
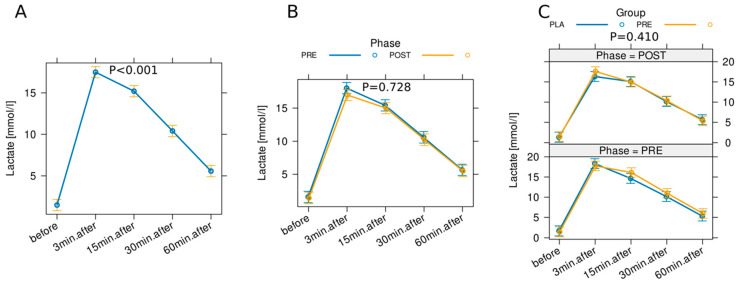
Blood lactate response to exercise before and after supplementation. Blood lactate response to exercise before and after supplementation. Panels show (**A**) the overall response across sampling times, (**B**) responses according to study phase (PRE vs. POST), and (**C**) the placebo (PLA) and probiotic (PRO) groups within each study phase. Within each panel, blood lactate concentration (mmol/L) is plotted at rest and at the post-exercise sampling times before (PRE) and after (POST) the four-week intervention. Points are estimated marginal means from the linear mixed-effects model (group × phase × sampling-time, with nested random intercepts for participant and participant-within-phase); error bars are 95% confidence intervals. The group × phase × sampling-time interaction was non-significant (χ^2^(4) = 6.65, *p* = 0.156), indicating that supplementation did not alter the exercise-induced blood lactate response.

The changes in blood LA concentration are presented in Figure 4. Exercise induced a pronounced increase in blood LA concentration at all post-exercise time points, with the peak observed at 3 min post-exercise, followed by a gradual decline at 15, 30, and 60 min (*p* < 0.001). However, lactate showed no group × phase (χ^2^(1) = 0.13, *p* = 0.715) or group × phase × sampling-time (χ^2^(4) = 6.65, *p* = 0.156) interaction; the pronounced exercise-induced rise (*p* < 0.001) was comparable between arms.

### 3.4. Effects of Supplementation on Muscle Damage Markers

**Figure 5:**
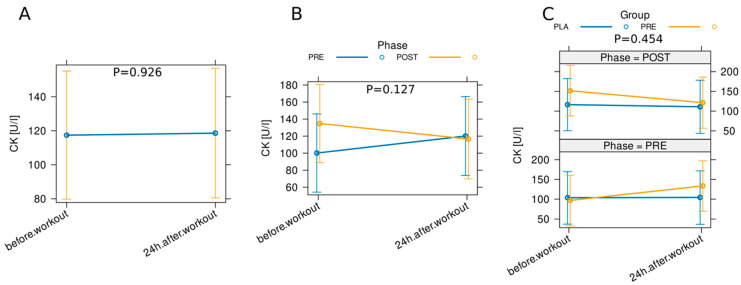
Creatine kinase (CK) response to exercise before and after supplementation. Panels show (**A**) the overall response across sampling times, (**B**) responses according to study phase (PRE vs. POST), and (**C**) the placebo (PLA) and probiotic (PRO) groups within each study phase. Within each panel, CK (U/L) is plotted before exercise and 24 h after exercise before (PRE) and after (POST) the four-week intervention. Points are estimated marginal means from the linear mixed-effects model (group × phase × sampling-time, with nested random intercepts for participant and participant-within-phase); error bars are 95% confidence intervals. The group × phase × sampling-time interaction was non-significant (χ^2^(1) = 1.42, *p* = 0.233), indicating that supplementation did not alter the exercise-induced CK response.

The CK responses are presented in Figure 5. Creatine kinase showed no group × phase (χ^2^(1) = 0.58, *p* = 0.448) or group × phase × sampling-time (χ^2^(1) = 1.42, *p* = 0.233) interaction between arms.

### 3.5. Adherence and Adverse Events

A total of 30 endurance runners were initially enrolled in the study, and 27 completed the full protocol. Three participants withdrew for personal reasons and therefore did not provide complete POST data. Mean capsule adherence, assessed by capsule count, was 92% in the probiotic (PRO) group and 88% in the placebo (PLA) group. Adherence to the standardized meal-box diet was monitored during follow-up interviews, and no major deviations from the prescribed dietary plan were reported. No serious adverse events related to the intervention were reported during the study period.

## 4. Discussion

The present study investigated whether four weeks of multistrain probiotic supplementation combined with a standardized diet could influence exercise performance, LA response, and inflammatory markers in trained endurance runners. No outcome showed a statistically significant group × phase interaction, and the group-related differences were small and non-significant. These findings suggest that, in well-trained athletes with relatively stable training and dietary pattern, short-term probiotic supplementation may not be sufficient to induce measurable physiological adaptations in these domains. Nevertheless, several outcomes showed small descriptive between-group differences that may warrant further investigation. Given the relatively small sample size and the large number of secondary outcomes, the study may have been underpowered to detect subtle intervention effects. Accordingly, these descriptive observations should be interpreted cautiously and regarded as exploratory rather than confirmatory.

Several factors may plausibly explain the absence of measurable effects. First, the four-week intervention may have been too short: microbiota remodeling and any downstream physiological adaptation typically require sustained exposure, and most trials reporting benefits used longer supplementation periods of 11–14 weeks [25]. Second, the delivered dose (2 × 10^9^ CFU/day) is at the lower end of the range used in ergogenic studies and may have been insufficient to establish durable colonization or metabolite production. Third, our participants were highly trained runners with well-consolidated aerobic phenotypes and stable training and nutritional habits; such athletes have limited adaptive headroom over four weeks, so any small effect would be difficult to detect against high baseline fitness. Finally, the fully standardized, fibre-defined diet—a deliberate strength of the design—may itself have constrained the substrate variability on which probiotic-driven microbial shifts depend, further limiting the scope for a detectable effect. These considerations, together with the modest sample size, suggest that the null result should be interpreted as absence of a demonstrable effect under these specific conditions rather than as evidence that probiotics are inert in endurance athletes.

Although no statistically significant effect of probiotic supplementation on inflammatory markers was detected in the present study, the available evidence suggests that probiotic supplementation may influence exercise-related immune and inflammatory responses under some conditions. IL-6 is one of the key cytokines released during prolonged or intensive exercise and is widely considered an indicator of the acute inflammatory and metabolic response associated with muscular work, as contracting skeletal muscle is one of the major sources of circulating IL-6 during exercise [26]. Potential mechanisms proposed in the literature include modulation of gut microbial activity and intestinal barrier function, which may in turn influence systemic immune signaling [15]. Because no microbial or metabolomic outcomes were measured, the mechanistic explanations discussed below are drawn from the existing literature and should be regarded as hypotheses rather than as inferences supported by our data. Nevertheless, findings from intervention studies in athletes remain inconsistent. For example, Lamprecht et al. reported that 14 weeks of multispecies probiotic supplementation in trained male endurance athletes reduced circulating TNF-α concentrations and zonulin levels, suggesting improved intestinal barrier function and reduced systemic inflammation [27]. Moreover, Martarelli et al. observed that 30 days of probiotic supplementation in trained endurance athletes, including runners and triathletes, was associated with a decrease in *C*-reactive protein (CRP) and markers of oxidative stress [28].

In contrast, West et al. found that 11 weeks of Lactobacillus fermentum supplementation in competitive cyclists did not significantly modify IL-6 or TNF-α responses to exercise despite periods of intensive training [5]. Similarly, Kekkonen et al. demonstrated that three months of Lactobacillus rhamnosus GG supplementation in endurance runners did not lead to significant changes in circulating IL-6, interleukin-10 (IL-10), or CRP concentrations during heavy training periods [8]. Comparable findings were reported by Harnett et al., who showed that 12 weeks of probiotic supplementation in trained athletes did not significantly alter post-exercise IL-6 or TNF-α blood concentrations, although some immune parameters were affected [6]. These findings are difficult to compare directly because the studies differ in probiotic strains, dose, supplementation duration (often 11–14 weeks versus four weeks here), athletic populations, co-supplements, and exercise protocols; such heterogeneity limits any firm cross-study inference.

The findings of the present study indicate that probiotic supplementation did not significantly affect VO_2_peak, TTE, or the majority of anaerobic performance variables measured during the Wingate test. Both total work and maximal power showed small increases in the two groups after the intervention period. This pattern most likely reflects continued training adaptation or a familiarization effect associated with repeated testing rather than a direct physiological effect of the supplementation itself. Mean power increased slightly in both groups over the intervention period (PLA: +0.11 W·kg^−1^; PRO: +0.17 W·kg^−1^). However, these descriptive improvements were not accompanied by a statistically significant group × phase interaction (*p* = 0.568) and therefore should be interpreted with caution, as they do not provide evidence of a probiotic supplementation effect.

Studies investigating the influence of probiotic supplementation on exercise performance have reported heterogeneous findings. Some investigations conducted in endurance athletes have demonstrated improvements in selected physiological and performance indicators. For example, Smarkusz-Zarzecka et al. reported that three months of supplementation with a multispecies probiotic in long-distance runners was associated with improvements in VO_2_peak, minute ventilation, and exercise capacity in male athletes [3]. Similarly, Huang et al. demonstrated that supplementation with *Lactiplantibacillus plantarum* TWK10 increased time to exhaustion and improved aerobic performance in physically active adults [29]. In contrast, other studies have not observed changes in classical performance indicators following probiotic supplementation. Schreiber et al. showed that 90 days of multispecies probiotic supplementation in elite road cyclists did not significantly alter VO_2_peak or time to fatigue, although participants reported lower perceived exertion during exercise testing [7]. In addition, systematic reviews suggest that the potential ergogenic effects of probiotics appear to depend largely on the specific microbial strains administered, the duration of supplementation, characteristics of the athletic population, and the type of exercise protocol used [9]. This point is particularly important when interpreting the present findings, as the available literature includes studies using single-strain and multispecies preparations, different supplementation periods, recreationally active individuals as well as elite athletes, and a wide range of exercise tests and performance outcomes. These methodological differences substantially limit direct comparisons with the current trial.

Our previous trial in mixed martial arts athletes provides a useful point of reference, but any comparison must be made cautiously. In that study, the same probiotic strains were co-administered with vitamin D_3_ over an identical period, and improvements were observed in selected performance parameters, including Wingate outcomes and prolonged TTE [18]. Crucially, because vitamin D_3_ was given alongside the probiotic, those effects cannot be attributed to the probiotic alone, and the combat-sport population, intermittent high-intensity metabolic profile, and uncontrolled diet differ substantially from the present continuous-endurance, diet-standardized design. These differences—in co-supplementation, strain exposure context, population, and exercise modality—mean that the divergent findings should not be interpreted as a direct contradiction. One important difference concerns dietary control. In this study, participants followed a standardized dietary regimen, which helped minimize nutritional variability that could influence gut microbiota composition and metabolic responses to exercise. Because diet is one of the key determinants shaping microbial activity in the gastrointestinal tract, controlling nutritional intake allows a more precise evaluation of the isolated effect of probiotic supplementation. In addition, the physiological demands of the investigated sports differ substantially. Combat sport disciplines typically involve intermittent bouts of high-intensity activity separated by short recovery intervals, whereas long-distance running is characterized predominantly by continuous aerobic exercise. Consequently, it is plausible that potential benefits of probiotic supplementation, particularly when combined with vitamin D_3_ may manifest differently depending on the metabolic profile and training demands of a given sport discipline.

In this study, the LA response to exercise was also evaluated. Blood LA concentration is widely used as an indicator of metabolic stress during physical effort and reflects the balance between LA production in working muscles and its transport and utilization in other tissues [30]. The intervention did not significantly affect LA concentrations measured either after the treadmill test to exhaustion or following the sprint effort. A comparable absence of changes in LA response was also reported in several previous studies conducted in athletes. For example, West et al. demonstrated that 11 weeks of Lactobacillus fermentum supplementation in competitive cyclists did not influence the metabolic response to exercise, including post-exercise LA concentrations [31]. Similarly, Schreiber et al. did not observe significant changes in exercise-related metabolic markers following 90 days of multispecies probiotic supplementation in elite road cyclists [7].

One explanation for these findings may lie in the fact that blood LA represents a systemic marker reflecting not only its production but also its transport and utilization across multiple tissues [32]. Consequently, metabolic effects associated with gut microbiota modulation may not necessarily translate into measurable differences in post-exercise LA concentrations. Recent studies indicate that gut microbiota may participate in the further metabolism of LA produced during exercise. Scheiman et al. demonstrated that bacteria from the genus Veillonella can utilize exercise-derived LA and convert it into propionate, which may subsequently serve as an additional energy substrate for the host [1]. These observations raise the hypothesis that any influence of gut microbiota on exercise metabolism might relate more to the efficiency of substrate utilization than to peak blood LA concentrations. We emphasize, however, that this interpretation is speculative in the context of the present study: we did not assess gut microbiota composition, intestinal permeability, SCFAs, or any microbial metabolite, and therefore cannot attribute our findings to any specific mechanism. Whether probiotic effects, if present, operate through bacterial metabolites such as SCFAs remains a hypothesis to be tested in studies that directly measure these variables [15,32].

## 5. Conclusions

In summary, four weeks of multistrain probiotic supplementation combined with a standardized diet did not produce statistically significant effects on aerobic capacity, anaerobic performance, lactate response, muscle-damage markers, or circulating inflammatory cytokines in this sample of trained male endurance runners. Within the limits of an exploratory trial of modest size, these findings indicate that short-term probiotic supplementation does not measurably alter the assessed physiological responses to exercise in athletes maintaining stable training and standardized nutritional conditions. Because gut microbiota composition and microbial metabolites were not measured, we make no claim regarding underlying mechanisms. Adequately powered trials with prespecified primary endpoints, longer supplementation, and direct microbiome and metabolomic assessment are needed to determine whether, and under what conditions, probiotic supplementation influences physiological responses to endurance exercise.

### Limitations of the Study

Several limitations should be considered when interpreting these findings. First, the relatively small sample size (27 completers) limited statistical power to detect small physiological effects, and the four-week intervention may have been too short to elicit measurable adaptations, particularly in systemic inflammatory markers. Second, although the standardized diet provided strict control over nutritional factors known to influence gut microbiota activity, we did not directly characterize gut microbial composition, intestinal permeability, or microbial metabolites such as short-chain fatty acids. Consequently, the proposed microbiota-related mechanisms remain speculative in this study, and integrating performance assessment with microbiome and metabolomic analyses would provide a more comprehensive mechanistic understanding. Third, training load was monitored using self-reported training diaries rather than objective, device-based load monitoring, which may have introduced measurement error. Fourth, only male athletes were enrolled to reduce sex- and menstrual-cycle-related variability; therefore, these findings should not be generalized to female endurance runners. Finally, the analysis was per-protocol (complete-case), based on the 27 participants who completed both assessments, so it does not follow the intention-to-treat principle. In addition, the trial was registered retrospectively (ClinicalTrials.gov NCT07411482; first posted February 2026) rather than prospectively, which is a further limitation. Future investigations involving larger cohorts, longer supplementation periods, direct microbiome and metabolomic profiling, objective training-load monitoring, and both sexes are warranted.

## Figures and Tables

**Table 1:** Blood and capillary sampling timeline during each assessment session (identical at PRE and POST).

Time Point (Relative to Protocol)	Stage	CK (Venous)	IL-6/TNF-α (Venous)	LA (Capillary)
Rest, pre-exercise (baseline)	Seated rest before testing	●	●	●
Immediately post-aerobic test	End of incremental treadmill test			●
15 min post-aerobic test	During passive recovery		●	●
3 min post-sprint	Recovery after Wingate			●
15 min post-sprint	Recovery			●
30 min post-sprint/post-protocol	End of protocol		● ^1^	●
60 min post-sprint	Recovery			●
24 h post-sprint	Next-day follow-up	●		

● = sample collected. ^1^ IL-6/TNF-α “30 min after completion of the exercise protocol” corresponds to 30 min post-sprint. CK, creatine kinase; IL-6, interleukin-6; TNF-α, tumor necrosis factor-α; LA, lactate.

**Table 2:** Participant characteristics.

Variable	PRO	PLA	*p* Value
	Mean ± SD	Mean ± SD	
Age	42.08 ± 6.82	39.43 ± 8.97	0.399
Height (cm)	179.77 ± 6.14	177.57 ± 6.84	0.389
Weight (kg)	76.76 ± 12.11	74.32 ± 8.37	0.545
FFM (kg)	63.74 ± 7.71	62.34 ± 7.04	0.627
FM (kg)	13.02 ± 5.57	11.99 ± 4.71	0.605
VO_2_peak (mL·kg^−1^·min^−1^)	55.40 ± 7.85	55.00 ± 7.60	0.896
HRmax (bpm)	178.62 ± 8.54	175.46 ± 9.57	0.384
Weekly training session (*n*/week)	5.07 ± 0.62	5.31 ± 0.63	0.335
Mean training session duration (min)	82.71 ± 3.79	84.54 ± 3.45	0.203
Mean training HR (bpm)	151.57 ± 1.91	152.62 ± 1.89	0.167
Mean training RPE	5.90 ± 0.24	5.96 ± 0.19	0.460

PRO—probiotic; PLA—placebo; SD—standard deviation; FM—fat mass; FFM—fat-free mass; HRmax—maximal heart rate obtained during VO_2_peak test; VO_2_peak—peak oxygen uptake obtained during aerobic sport test; RPE—rating of perceived exertion.

**Table 3:** Effects of supplementation on aerobic and anaerobic performance: descriptive PRE/POST means per arm and the model-based between-group difference-in-change (group × phase interaction).

Variable	PLA PRE	PLA POST	PRO PRE	PRO POST	Between-Group Δ-In-Change (PRO − PLA) [95% CI]	*p*	Cohen’s d
Aerobic fitness							
VO_2_peak (mL·kg^−1^·min^−1^)	55.00 ± 7.60	57.33 ± 8.41	55.40 ± 7.85	53.14 ± 5.95	−3.54 [−7.01, −0.07]	0.044 ^1^	−0.75
RER (VCO_2_/VO_2_)	1.16 ± 0.10	1.19 ± 0.07	1.12 ± 0.05	1.13 ± 0.04	−0.015 [−0.081, 0.051]	0.658	−0.12
VE (L·min^−1^)	150.69 ± 27.86	150.46 ± 26.96	149.92 ± 12.92	149.77 ± 12.49	+0.41 [−10.79, 11.61]	0.943	+0.04
TTE (s)	1196.62 ± 160.01	1211.54 ± 126.00	1212.10 ± 102.84	1212.70 ± 91.41	−6.45 [−71.86, 58.96]	0.847	−0.05
Anaerobic fitness							
Wtot (J·kg^−1^)	164.29 ± 16.67	166.34 ± 17.78	162.13 ± 20.27	165.53 ± 17.59	+1.35 [−3.07, 5.77]	0.549	+0.23
MP (W·kg^−1^)	8.21 ± 0.83	8.32 ± 0.89	8.11 ± 1.01	8.28 ± 0.88	+0.064 [−0.156, 0.285]	0.568	+0.22
Pmax (W·kg^−1^)	9.54 ± 1.13	9.66 ± 1.14	9.32 ± 1.23	9.44 ± 1.09	+0.005 [−0.331, 0.342]	0.976	+0.01
Time-to-Pmax (s)	5.99 ± 0.94	5.81 ± 1.28	6.52 ± 1.93	5.71 ± 0.90	−0.63 [−1.84, 0.58]	0.305	−0.39

Values are mean ± SD. Between-group Δ-in-change = model-based group × phase interaction (difference between arms in the PRE → POST change) from linear mixed-effects models with a participant random intercept. *p* = nominal *p*-value for the interaction. ^1^ VO_2_peak did not remain significant after Benjamini–Hochberg FDR correction across outcomes (adjusted *p* = 0.707). Cohen’s d computed on change scores (pooled SD). Descriptive means use all available data at each time point. The mixed models retained all available observations under a missing-at-random assumption; the missing VO_2_peak data occurred in the placebo arm—one participant lacked a PRE value and a different participant a POST value—so PLA contributed *n* = 13 per time point (12 complete PRE → POST pairs), whereas the probiotic arm was complete (*n* = 13). CI—confidence interval; FDR—false discovery rate; MP—mean power; PLA—placebo; Pmax—maximal power; POST—after supplementation; PRE—before supplementation; PRO—probiotic; RER—respiratory exchange ratio obtained in aerobic sport test; SD—standard deviation; TTE—time to exhaustion; VE—minute ventilation; VO_2_peak—peak oxygen uptake obtained during aerobic sport test; Wtot—total work.

## Data Availability

The data presented in this study are available on request from the corresponding author due to privacy or ethical restrictions.

## Abbreviations

The following abbreviations are used in this manuscript:

PRE	Before intervention
POST	4 weeks after intervention
PRO	probiotic
PLA	placebo
FFM	Fat-free mass
FM	Fat mass
TTE	time to exhaustion
RPE	subjective ratings of perceived exertion
HR	heart rate
IL-6	Interleukin-6
TNF-α	tumor necrosis factor alpha
LA	Lactate
CK	Creatine kinase
MP	Mean power
P_max_	Maximal power
VO_2_peak	Peak oxygen uptake
RER	Respiratory exchange ratio
VE	minute ventilation
W_tot_	Total work
